# Anatomical Study of the Neurovascular in Flexor Hallucis Longus Tendon Transfers

**DOI:** 10.1038/s41598-017-13742-0

**Published:** 2017-10-27

**Authors:** Haijiao Mao, Wenwei Dong, Zengyuan Shi, Weigang Yin, Dachuan Xu, Keith L. Wapner

**Affiliations:** 1 Department of Orthopaedic Surgery, Affiliated Hospital of the School of Medicine of Ningbo University, No. 247, Renming Road, Jiangbei District, Ningbo, Zhejiang, China; 2 Department of Anatomy, Medical School of Ningbo University, Ningbo, Zhejiang, China; 3 Department of Anatomy, Southern Medical University, No. 1023 Shatai nan Road, Guangzhou, Guangdong, China; 4 Department of Orthopaedic Surgery, University of Pennsylvania, Philadelphia, PA USA

## Abstract

The transfer of the flexor hallucis longus tendon or flexor digitorum longus tendon is frequently used for the treatment of posterior tibial tendon insufficiency or chronic Achilles tendinopathy. According to several anatomical studies, harvesting the flexor hallucis longus (FHL) tendon may cause nerve injury. Sixty-eight embalmed feet were dissected and anatomically classified to define the relationship between Henry’s knot and the plantar nerves. Two different configurations were identified. In Pattern 1, which was observed in 64 specimens (94.1%), the distance between the medial plantar nerve and Henry’s knot was 5.96 mm (range, 3.34 to 7.84, SD = 1.12). In Pattern 2, which was observed in 4 specimens (5.9%), there was no distance between the medial plantar nerve (MPN) and Henry’s knot. No statistically significant difference was observed according to gender or side (p > 0.05). A retraction was performed to harvest the FHL through the posteromedial hindfoot incision using a single minimally invasive technique, and the medial and lateral plantar nerve lesions were scrupulously assessed. In conclusion, medial and lateral plantar nerve injuries did not occur more frequently, even after performing a single minimally invasive incision to harvest the FHL tendon, due to the large distance between the FHL tendon and the medial and lateral plantar nerves.

## Introduction

A flexor hallucis longus (FHL) tendon transfer is frequently used for the treatment of both posterior tibial tendon insufficiency and chronic Achilles tendinopathy^1–5^. Multiple surgical procedures to harvest the FHL have been described^6–10^. Autogenous FHL transfers have recently become among the most popular methods of augmenting Achilles or posterior tibial tendon reconstruction. The FHL muscle is stronger than other muscles, and its axis of contractile force most closely approximates that of the Achilles^7^ or posterior tibial tendon^2^.

Different techniques for harvesting the FHL tendon have been described. Originally, Hansen described harvesting the tendon through a single posterior-medial incision^6^. To harvest a longer tendon for transfer, Wapner *et al*. described a double-incision technique utilizing a medial midfoot and posterior-medial incision to harvest the FHL tendon^7^. Alternatively, the FHL tendon can be harvested using a posterior-medial minimally invasive technique at the plantar first interphalangeal (IP) joint to obtain additional length^11,12^. The location of the medial plantar nerve (MPN) and the lateral plantar nerve (LPN) near the decussation of Henry’s knot suggest that the FHL and FDL(flexor digitorum longus) could be at risk with this technique due to the lack of the direct exposure necessary for safely visualizing and identifying the structures located deep within the midfoot.

Injury to the nerves is a serious complication that leads to persistent pain and a poor clinical result^13,14^. Harvesting the FHL tendon via a transection performed distally to Henry’s knot may cause injuries to the medial or lateral plantar nerves. Although the anatomy of the plantar nerves has been described in cadaveric feet^15,16^, the precise anatomical relationship between Henry’s knot and the plantar nerves has not been reported. The anatomical relationship between Henry’s knot and the tendon length available for harvest varies across different ethnic or racial groups^15,17–19^. Therefore, we posit that similar variations between the plantar neurovascular bundle and Henry’s knot are likely. To the best of our knowledge, to date, no anatomical studies have been performed in Asian populations.

The purpose of the study is to describe the anatomical variations in the relationship between Henry’s knot and the plantar nerves in Asian specimens, introduce a thorough anatomical classification and quantify the distance between Henry’s knot and the plantar nerves. This information could inform surgeons of the incidence and communicating branching patterns of the plantar nerves, arteries and tendons in our population based on their anatomical relationship. This information could enable surgeons to avoid injury to these neurovascular structures.

## Results

In all 68 cadaver specimens, the plantar neurovascular structures and Henry’s knot could be exposed through a plantar incision, allowing the surgeon to cut the end of the FHL to be harvested and retracted into the hindfoot wound. The anatomical features of the communicating plantar neurovascular branches and Henry’s knot, including the shape, course, and anastomoses, were defined and classified.

### Bifurcation of vascular structure and nerve

**Figure 1:**
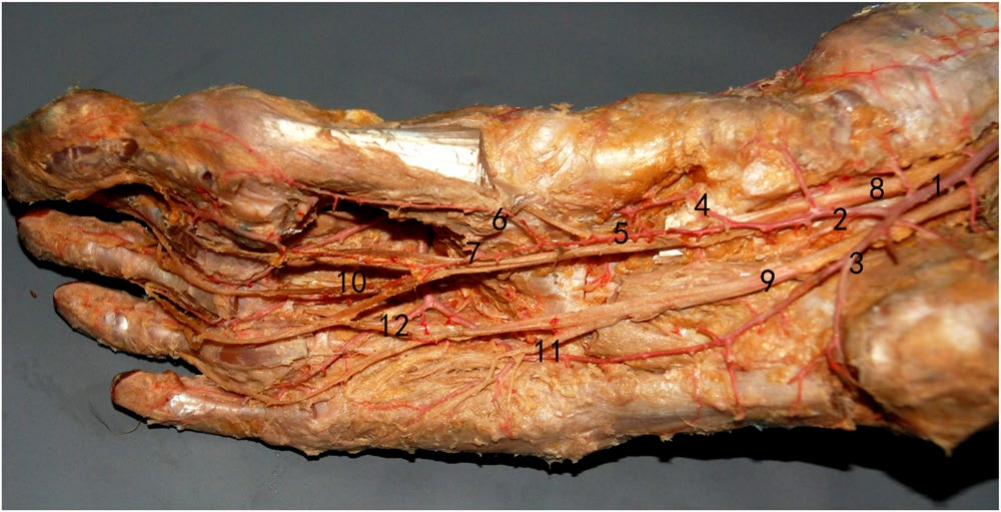
Bifurcation of the vascular structure and nerve (1). posterior tibial artery, (2). medial plantar artery, (3). lateral plantar artery, (4). superficial division of medial plantar artery (5). deep division of medial plantar artery, (6). Medial branch of deep division of medial plantar artery, (7). lateral branch of deep division of medial plantar artery, (8). medial plantar nerve, (9). lateral plantar nerve (10). common plantar digital nerve, (11). muscular branch to the abductor digiti quinti, (12). communicating branch.

After providing a branch to the heel, the posterior tibial artery divides into the medial and lateral plantar arteries. The medial plantar artery runs on the deep surface of the plantar aponeurosis between the abductor hallucis and the flexor digitorum brevis. The superficial division of the medial plantar artery pierces through the abductor hallucis around the navicular tubercle to the superficial layer. The deep division of the medial plantar artery runs between the abductor hallucis and flexor hallucis brevis muscles, dividing into the medial and lateral branches and the arterial arch of the medial foot. The external caliber of the deep division of the medial plantar artery was 0.8 ± 0.2 mm. The deep division of the medial plantar artery into the medial and lateral branches at occurred 38 ± 10 mm distal to the origin of the medial plantar artery. The medial branch of the deep division of the medial plantar artery runs between the abductor hallucis and the flexor hallucis brevis muscles (Fig. 1). The external caliber was 0.8 ± 0.2 mm. The lateral branch of the deep division of the medial plantar artery runs in the space between the first and second metatarsal below the plantar fascia. The external caliber was 0.9 ± 0.2 mm.

### Relationship between neurovascular structure and Henry’s knot

**Figure 2:**
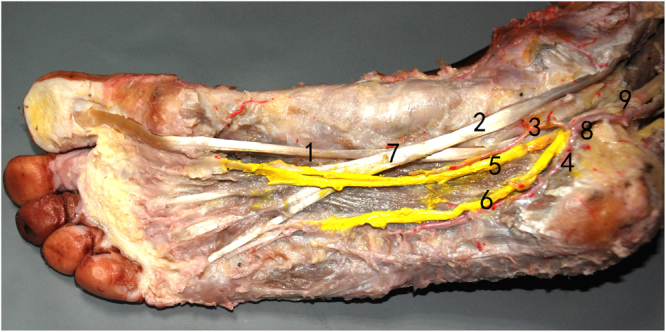
The distance between the medial plantar nerve and Henry’s knot in Pattern 1 (1). FHL, (2). FDL, (3). medial plantar nerve, (4). lateral plantar nerve, (5). medial plantar artery, (6). lateral plantar artery, (7). Henry’s knot, (8). posterior tibial artery, (9). tibial nerve.

**Figure 3:**
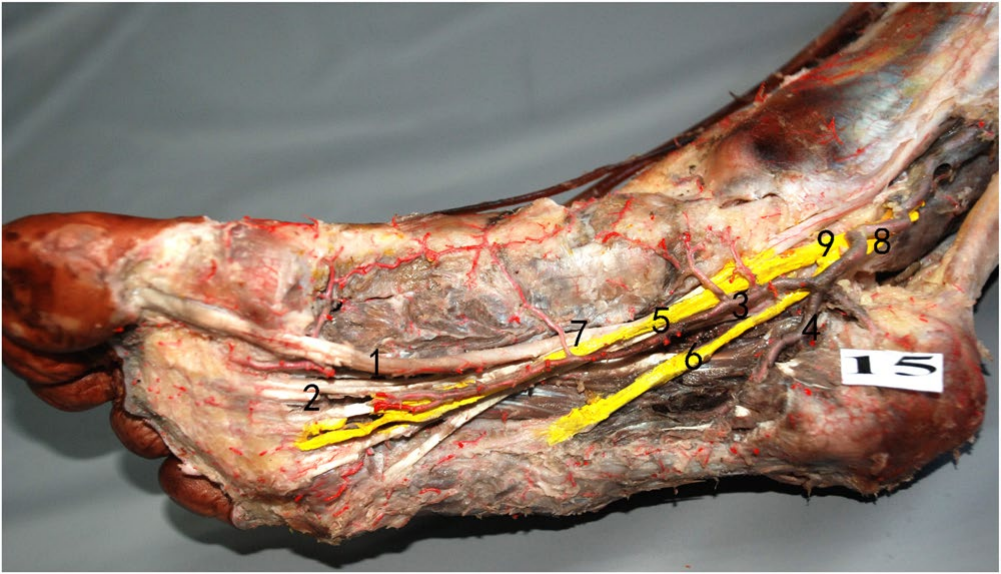
No distance between the medial plantar nerve and Henry’s knot in Pattern 2 (1). FHL, (2). FDL, (3). medial plantar nerve, (4). lateral plantar nerve, (5). medial plantar artery, (6). lateral plantar artery, (7). Henry’s knot, (8). posterior tibial artery, (9). tibial nerve, (10). artery.

The MPN is the largest and most anterior of the terminal branches of the TN. The MPN has muscular, cutaneous, articular and vascular branches and is responsible for the sensory innervation of most plantar aspects of the foot. The medial plantar nerve appears between the abductor hallucis and the flexor digitorum brevis. The MPN extends medial proper plantar digital nerve to the hallux and divides into three common plantar digital nerves near the metatarsal bases. These digital nerves pierce between the slips of the plantar aponeurosis and divide into two proper plantar digital nerves. The anatomical relationship between Henry’s knot and the plantar nerves was observed. Two patterns were identified. In Pattern 1, which was observed in 64 specimens (94.1%), the distance between the medial plantar nerve and Henry’s knot was 5.96 mm (range, 3.34 to 7.84, SD = 1.12) (Fig. 2). In Pattern 2, which was observed in 4 specimens (5.9%), there was no distance between the MPN and Henry’s knot (Fig. 3). The mean distance between the MPN and Henry’s knot was 5.26 mm. The LPN accompanied the lateral plantar artery into the plantar midfoot and demonstrated a distance of 15.5 mm (range, 8 to 27.8, SD = 4.2) from Henry’s knot. For data showing a normal distribution, the groups were compared by gender by performing an independent samples *t-*test. A paired samples *t-*test was performed to compare the means of the left and right sides. No statistically significant difference was observed in the distance by gender and side (Tables 1 and 2). Finally, the medial and lateral plantar nerves, particularly the medial plantar artery and nerves, were scrupulously assessed to identify their relationship with Henry’s knot.

**Table 1:** Comparison between the genders (male = 19; female = 15)

	male	female	p-value
MPL (mm)	6.28 ± 1.22	5.02 ± 1.22	0.12
LPN (mm)	16.4 ± 4.2	15.3 ± 5.2	0.17

MPN = medial plantar nerve

LPN = lateral plantar nerve

The p-values were determined using independent sample t-tests. Significance levels are indicated by p < 0.05.

**Table 2:** Comparison between the sides (n = 34)

	Left	right	*p-*value
MPL (mm)	6.04 ± 1.00	5.02 ± 1.18	0.36
LPN (mm)	16.5 ± 15.5	13.4 ± 12.3	0.43

MPN = medial plantar nerve

LPN = lateral plantar nerve

The p-values were determined using paired sample t-tests. Significance levels are indicated by p < 0.05.

**Figure 4:**
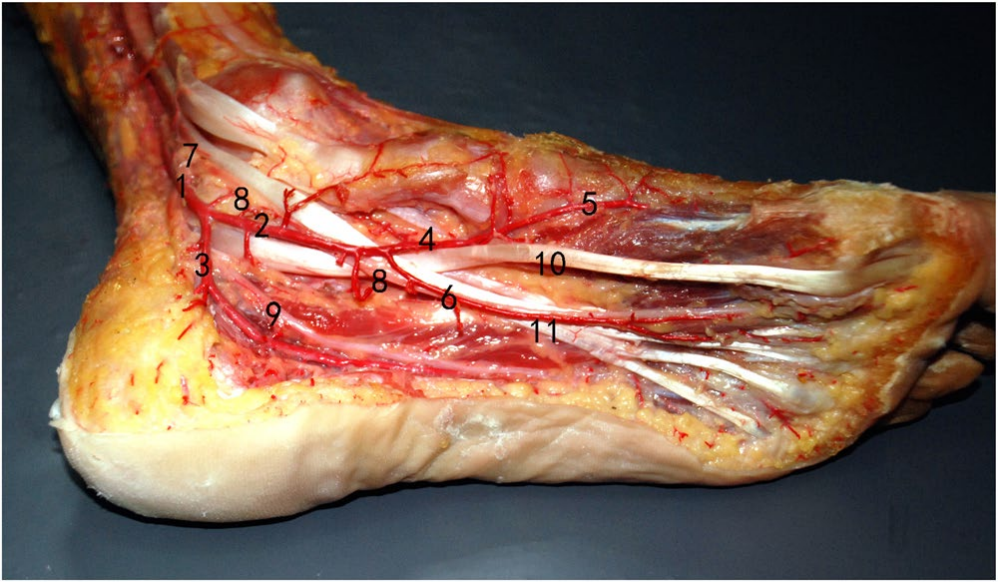
Relationship between the medial plantar artery and tendon (1). posterior tibial artery, (2). medial plantar artery, (3). lateral plantar artery, (4). superficial division of the medial plantar artery, (5). arterial arch at the superior border of the abductor hallucis, (6). deep division of the medial plantar artery, (7). tibial nerve, (8). medial plantar nerve, (9). lateral plantar nerve, (10). FHL tendon,(11). FDL tendon.

The superficial division of the medial plantar artery travels over the FHL, pierces through the abductor hallucis around the navicular tubercle to the superficial layer, and then proceeds distally into the formation of the arterial arch at the superior border of the abductor hallucis. The deep division of the medial plantar artery accompanies the MPN and is found in close proximity to the FDL tendon (Fig. 4).

### Relationship between the FHL and FDL in Henry’s knot

**Figure 5:**
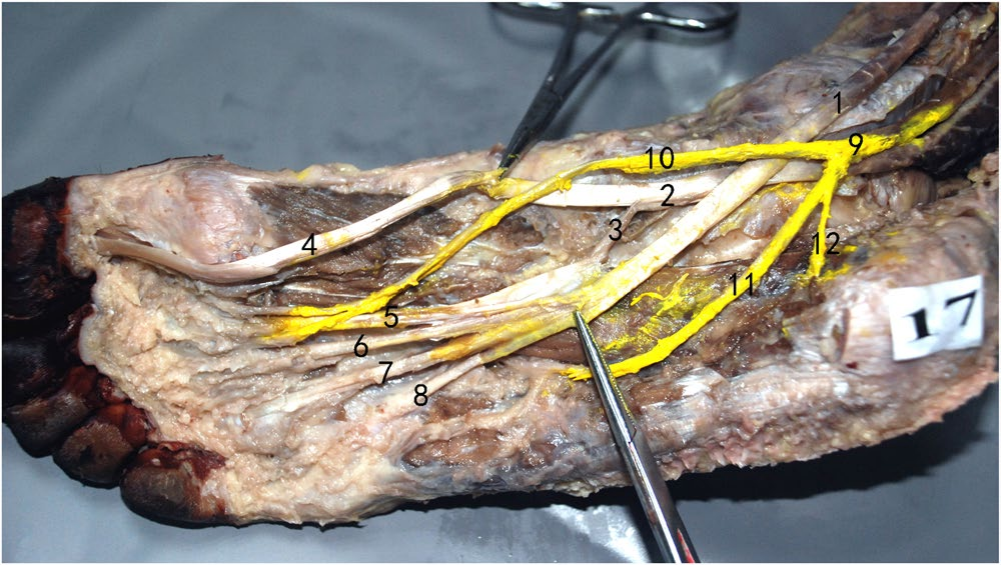
Type I (1). FDL tendon, (2). FHL tendon, (3). a tendinous slip is directed from the FHL tendon to the FDL tendon, (4). first (1^st^) long flexor tendon, (5). second (2^nd^) long flexor tendon, (6). third (3^rd^) long flexor tendon, (7). fourth (4^th^) long flexor tendon, (8). fifth (5^th^) long flexor tendon, (9). tibial nerve, (10). medial plantar nerve, (11). lateral plantar nerve, (12). calcaneal branch.

**Figure 6:**
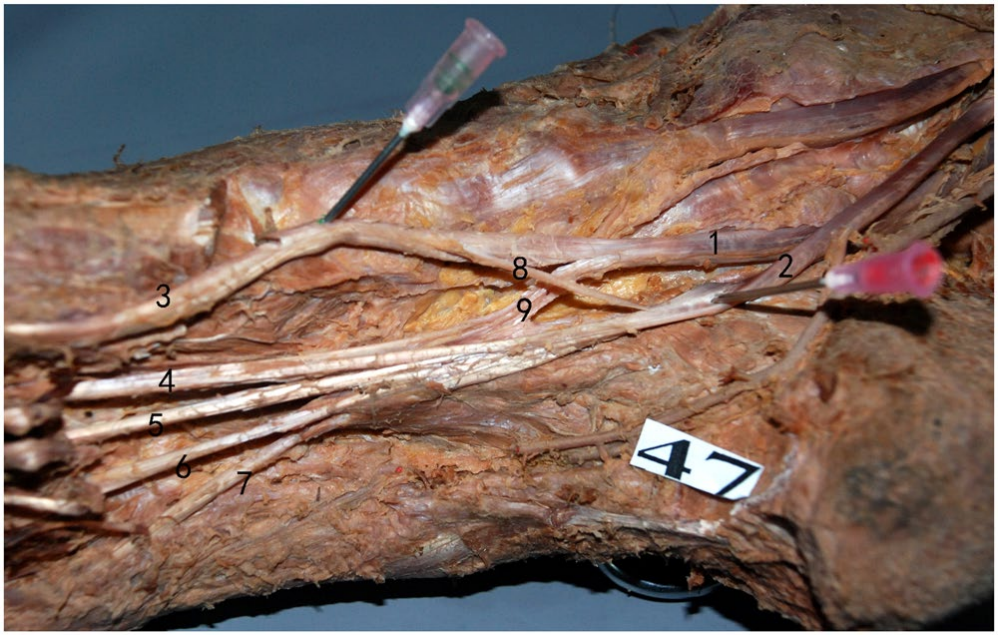
Type II (1). FHL tendon, (2). FDL tendon, (3). first (1^st^) long flexor tendon, (4). second (2^nd^) long flexor tendon, (5). third (3^rd^) long flexor tendon, (6). fourth (4^th^) long flexor tendon, (7). fifth (5^th^) long flexor tendon, (8). tendinous slip from FDL to FHL, and (9). tendinous slip from FHL to FDL.

Any cross-attachments between the FHL and FDL tendons were exposed and observed. The only two configurations of the distal relationship between the FHL and FDL were identified at Henry’s knot. In the Type I configuration, which was observed in 66 specimens, a single cross-attachment tendon slip directed from the proximal FHL tendon into the distal FDL tendon was observed (Fig. 5). In the Type II configuration, which was observed in 2 specimens, a tendon slip directed from the FHL to the FDL and a tendon slip directed from the FDL to the FHL were observed (Fig. 6). No other patterns were observed. The transection of these cross-attachments was required to harvest the FHL and retract the FHL into the posterior incision.

## Discussion

Transfer of the FHL tendon is frequently used for the treatment of both posterior tibial tendon insufficiency and chronic Achilles tendinopathy. To harvest and mobilize the FHL for transfer, exact knowledge of these interconnections, their position and relationship with the arterial and nerve structures and anatomical variations is essential. Surgeons should harvest the FHL tendon using a minimally invasive technique, which is important to assess the functional loss after graft. The anatomical relationship between the FHL and FDL tendons in Henry’s knot has been described in the surgical and anatomical literature^15,17,19–21^. Only two different configurations between the FHL and FDL tendons in Henry’s knot were identified in our previous reports^17^.

Few articles describing the complications of FHL tendon transfer have been published^15^. Certain patients with adult flatfoot deformity experienced paresthesias in the medial plantar nerve following treatment with calcaneal osteotomy and tendon transfer. Nerve injuries may lead to sensory disturbances and diminished motor function. However, Coull *et al*. reported the presence of postoperative morbidity due to the loss of FHL function following FHL tendon transfer for the treatment of chronic Achilles tendon rupture or chronic Achilles tendinosis, but no clinically significant neurological morbidity was identified^22^. The occurrence of nerve injury during FHL tendon harvest has been described in a cadaver study^15^. Injuries were observed in a large percentage of foot specimens (33%, eight feet), either to the medial plantar nerve (25%, six feet) or the lateral plantar nerve (8%, two feet). Similar clinical symptoms have been described after harvesting the FHL tendon. A previous series using a medial approach to Henry’s knot described an injury to the medial plantar nerve that occurred following FHL transfer performed for the re-rupture of the Achilles tendon^9^.

Due to the tendon cross-attachments present in Henry’s knot, the retraction of the FHL tendon may be difficult to achieve regardless of whether a double- or, single-incision technique or a minimally invasive incision technique is used for the FHL tendon harvest. The proximity of the MPN and LPN to the decussation of the FHL and FDL creates a vulnerable anatomical area in Henry’s knot. These observations led to the concern that the difficult retraction process could cause nerve injury^15^. Tashjian *et al*.^23^ mentioned the possibility of neurovascular injury while dissecting near the neurovascular bundle in Henry’s knot using a double-incision technique. With minimally invasive incisions, the medial plantar nerve is not consistently visualized^11^. The retraction of the FHL tendon into the posterior incision is difficult to achieve due to the presence of cross-attachments with the FDL tendon. The MPN and LPN may be injured because of their relationship to the tendons at Henry’s knot.

Injuries to the nerve and the accompanying vessels can be reliably avoided during the FHL transfer with a careful understanding of the anatomical relationships^9^. When performing an FHL transfer for Achilles tendinopathy using a single-incision approach, the tendon is harvested via a posterior-medial incision at the level of the Achilles tendon. The FHL tendon is transected behind the ankle joint, proximal to Henry’s knot, and nerve injury should be avoided, but the length of the tendon available for transfer is much shorter.

To the best of our knowledge, no previous study has quantified the relationship between Henry’s knot and the plantar nerves. The results of this study confirm two patterns of relationships between Henry’s knot and the plantar nerve and arteries. Cross-attachments between the FHL and FDL tendons were present in all foot specimens.

The defined distance between the medial plantar nerve and Henry’s knot was present in most specimens. The medial planter nerve overlapped Henry’s knot in only 4 specimens. Clinically, extensive dissection and a large exposure of the FHL and FDL tendons are unnecessary using a double-incision approach for FHL tendon harvest. A larger incision may lead to an increased risk of infection or other postoperative morbidities^24^. Neurological or vascular injuries are clinically infrequent, which is consistent with the results of this anatomical study.

Particularly in Pattern 2, the FHL tendons should be separated carefully, and the subsequent retraction of the FHL tendon should be performed gently to avoid a partial or complete rupture of the medial plantar nerve. The lateral plantar nerve and artery do not appear to be at risk due to the distance between these structures and Henry’s knot.

Nerve injuries can lead to sensory disturbances and diminished motor function. The MPN runs along the plantar surface of the FDL tendon and through Henry’s knot. The MPN continues at the medial border of the foot and branches medially and laterally. The MPN supplies sensation to the first through fourth toes and innervates the abductor hallucis, flexor hallucis brevis, flexor digitorum brevis, and lumbrical muscles. Overlap and variations exist in this division, and the communicating branch between the lateral and medial plantar nerves can further explain the variations in the plantar digital sensory patterns. A communicating branch was present between the third and fourth intermetatarsal-space nerves in 28% of all foot specimens^16^. However, Frank *et al*.^25^ reported a communicating branch that was present in 66.2% of the feet that showed no large gender-based differences. Govsa *et al*.^16^ reported a perpendicular coursing communicating branch that could be at a higher risk of being severed during surgery. The fascicular patterns of the LPN, MPN and CB groups showed no interfascicular communication in our study. Plantar sensory disturbances caused by MPN injury during the harvesting of the FHL tendon cannot be compensated for by the LPN.

Most foot specimens demonstrated a large distance between the medial plantar nerve and Henry’s knot, which is consistent with the previously reported low incidence of related clinical symptoms. Furthermore, partial lesions may not be sufficient to cause clinical symptoms.

When a single-incision technique is used, the incision is performed posterior medial to the Achilles tendon, and the transection of the FHL tendon occurs proximally to Henry’s knot at the level of the posterior ankle. This technique reduces the risk of direct nerve injury to the medial and lateral plantar nerves but is limited compared to double-incision techniques that offer a greater tendon graft length for the repair of larger tendon defects than that offered by a single incision. The double-incision technique for harvesting the FHL tendon, which was first described by Wapner *et al*., allows tenodesis of the distal FHL remnant to the FDL^26^. This technique requires more extensive dissection to transect the FHL tendon distal to Henry’s knot. Any cross-attachments should be exposed and carefully transected before retraction is performed. To avoid these complications, several authors have used minimally invasive incision techniques to harvest the FDL and FHL using direct plantar approaches^9,12^.

Our previous results demonstrate that compared with a single-incision technique, on average, an additional 17 mm of tendon graft are obtainable using the double-incision technique if the FHL is transected at Henry’s knot^17^. This result is considerably lower than the 100–120 mm of additional tendon length previously reported by Wapner *et al*.^26^. The difference in length may be due to the more distal transection site at the midshaft of the first metatarsal or ethnic differences. Compared with the double-incision technique with a resection at Henry’s knot, on average, an additional 110 mm of tendon graft are obtainable using a minimally invasive technique. Based on these results, we posit that different incision techniques should be chosen according to the size of the Achilles tendon defect.

Based on our data, we do not recommend harvesting the FHL tendon at Henry’s knot because the proximity of the MPN may lead to injury, and the length of the tendon available is shorter than that when harvesting at the midshaft of the first metatarsal or the first IP joint. When approaching the tendon distal to Henry’s knot, the dissection of the interconnecting fiber is crucial for harvesting the FHL; thus, the surgeon must have precise knowledge regarding the tendinous variations in the region of Henry’s knot. In our study, we found that most cases, i.e., 66 of 68 specimens (97.1%), have cross-attachment directed from the FHL to the FDL. In contrast, the presence of cross-attachments in both directions was rare and only observed in 2 of the 68 specimens (2.9%). The minimally invasive technique of using a smaller forefoot incision to harvest the FHL tendon may provide more tendon more quickly, which will is particularly suitable for large tendon defects.

Nevertheless, this study has some limitations. All specimens were from an Asian population, and therefore, possible ethnic or racial differences could not be analyzed. The results of this study are limited to cadavers, and the relative stiffness of cadaver tendons may have influenced the results. Although the feet were inspected for previous surgery or diseases of the foot and were in good condition, we had no knowledge of any medical history regarding foot and ankle problems. The embalming of the specimen could have led to changes in the examined tissues due to tissue shrinkage, which may have also led to errors in the measurements of the distance of the relationships.

Furthermore, we only considered the FHL tendon, and the FDL tendon was not measured or anatomically classified. Several studies support the choice of the FDL tendon for the treatment of both posterior tibial tendon insufficiency and chronic Achilles tendinopathy^27–32^. Thus, exact knowledge of the complicated anatomical relationship between the nerves and the tendon at Henry’s knot should assist surgeons in avoiding injury to the plantar nerves when harvesting the FHL or the FDL tendon at this level.

Based on the present data, comprehensive knowledge of the anatomical relationship between the plantar nerves and Henry’s knot is essential to safely perform FHL tendon harvest. We believe that a minimally invasive incision technique is feasible and may be less likely to cause injury to the nearby tibial, medial plantar, and lateral plantar nerves, particularly for large tendon defects. While the plantar area remains an important anatomical site for clinicians, further anatomical studies are warranted. Clinical studies evaluating this minimally invasive incision technique compared with the currently used double-and single-incision techniques in terms of the associated morbidity and nerve injury are needed.

## Materials and Methods

The experiment was conducted in collaboration with the Department of Anatomy at Ningbo University and Southern Medical University in accordance with the Declaration of Helsinki (World Medical Association). Informed consent was obtained from the families who participated in the donation program at Southern Medical University and Ningbo University. This study was approved by the Ethics.

Committee of the Affiliated Hospital of the School of Medicine of Ningbo University. The cadaver legs were donated to the anatomy laboratory of the Medical School of Southern Medical University and Ningbo University. Sixty-eight legs from 34 cadavers were analyzed anatomically with respect to the relationship between Henry’s knot and the plantar nerves in feet without obvious deformity or evidence of previous surgery and dissection. The cadavers’ mean age was 65.2 (range from 14 to 82) years. Thirty-eight feet were from 19 male cadavers, and thirty feet were from 15 female cadavers; the cadavers were embalmed with a mixture of ethanol, formaldehyde, glycerin, phenol and parachlorometaxylenol. The cadavers were subsequently fixed by immersion in a formalin solution for at least 1 year. The popliteal arteries were dissected, and red latex was manually injected under physiological pressure with a syringe until the skin of the toe became red. The latex was allowed to cure overnight at room temperature.

The skin, superficial fascia, and plantar aponeurosis were removed. The dissection was performed under a dissection microscope to meticulously delineate the branches of the posterior tibial artery and tibial nerve. Then, the muscles of the flexor digitorum brevis, flexor hallucis brevis, abductor hallucis and lumbricals were meticulously removed. The posterior tibial tendon, FDL tendon, and FHL tendon were identified and exposed.

The navicular tubercle, sustentaculum tali and first interphalangeal joint were marked on the medial foot. The FHL and FDL tendons were dissected and exposed in their course from the point of the musculotendinous junction to their distal insertion. The FHL and FDL tendons were exposed at the level of Henry’s knot. Any cross-attachments between the FHL and FDL tendons were identified, counted and classified. The functional insertion of the interconnections of the FHL to the FDL was analyzed. Tension was manually applied to the FDL and FHL tendons individually near the region of Henry’s knot, and digit movement was recorded.

**Figure 7:**
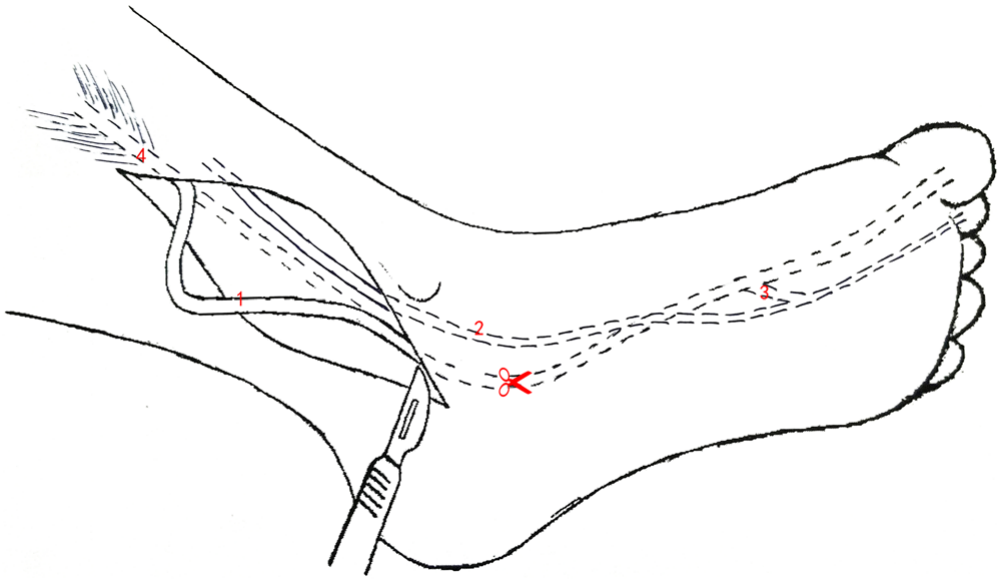
A single-incision technique (1). FHL tendon, (2). FDL tendon, (3). tendinous slip, (4). musculotendinous junction of FHL The red scissors indicate that the FHL tendon was cut at the level of the sustentaculum tali.

**Figure 8:**
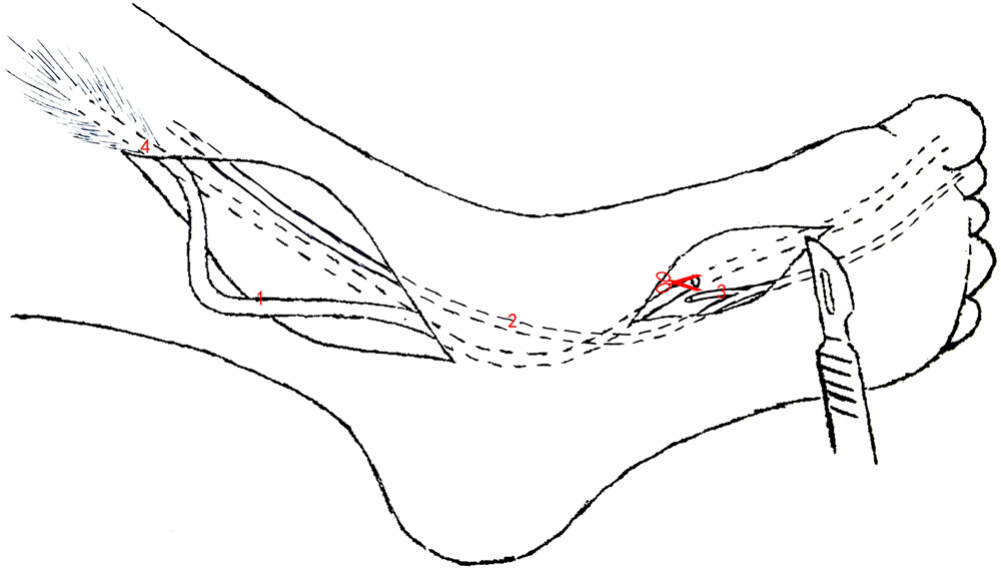
Double-incision technique (1). FHL tendon, (2). FDL tendon, (3). tendinous slip, (4). musculotendinous junction of FHLThe red scissors indicate that the FHL tendon was cut at the level of Henry’s knot.

**Figure 9:**
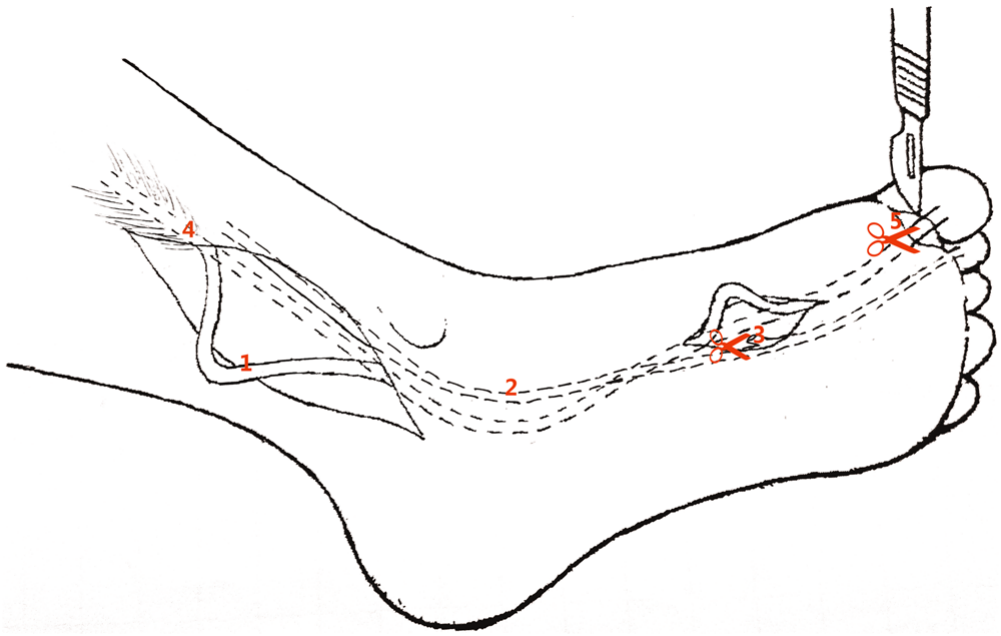
Minimally invasive technique (1). FHL tendon, (2). FDL tendon, (3). tendinous slip, (4). musculotendinous junction of FHL, and (5). first interphalangeal (IP) joint The red scissors indicate that the FHL tendon was cut at the level of IP joint. The tendon slip was cut in Henry’s knot via a small incision midfoot. The FHL tendon was pulled from the posterior-medial incision.

A single-incision technique was used to harvest the FHL tendon through a single posterior-medial incision (Fig. 7). The distance between the musculotendinous junction of the FHL and the point at the sustentaculum tali was calculated and represented the length of the FHL tendon for harvesting through a single posterior-medial incision. Compared to a single-incision technique, an additional small incision in the midfoot was necessary for tendon graft using a double-incision (Fig. 8). The distance between the musculotendinous junction of the FHL and the level of Henry’s knot was measured to represent the available FHL graft from the double-incision technique. The minimally invasive incision technique required another two small incisions over the midfoot and first IP joint. The FHL tendon was cut in these two incisions and then pulled from the posterior-medial incision (Fig. 9). The length from the musculotendinous junction of the FHL to the first IP joint was measured to represent the available FHL graft with minimally invasive incision.

The tendinous connections between the FHL and FDL were classified using a modified classification system^19,20^. Type I describes an attachment branching from the FHL tendon proximally to the FDL tendon Type II describes a crossed connection; Type III describes a proximal to distal connection of the FDL to the FHL; and Type IV describes no connection between the tendons.

The posterior tibial nerve, posterior tibial artery and its branches were dissected and exposed from the medial and lateral plantar nerve origins to the level of this region. The dissection was performed until adequate exposure of the posterior tibial nerve ramifying in the medial and lateral plantar branches was achieved. The branches of the posterior tibial artery were identified by injecting red latex. To distinguish the arteries from the nerves, all nerves were sprayed with lead nitrate and potassium dichromate and became yellow.

The nerves were carefully assessed to identify any neurological injury that occurred during the FHL harvest with different incisions. The distance between the tendons and the medial and lateral plantar nerves at Henry’s knot was measured in millimeters using a slide gauge. We also measured the external diameters of all arteries and observed the anastomoses between the arteries. This area was photographed in all specimens using a digital camera (Nikon D-80). The anatomical features of Henry’s knot and the plantar nerves were classified. When the FHL was retracted into the posteromedial hindfoot incision, the medial and lateral plantar nerves lesions were scrupulously assessed.

The statistical analysis was conducted using the Statistical Package for the Social Sciences (SPSS, IBM), version 20.0. The statistical significance level was defined as *p* < 0.05. The Kolmogorov-Smirnov test was performed in the distribution of cases. For data showing a normal distribution, the groups were compared by gender using an independent samples *t-*test. Paired samples t-tests were performed to compare the means of the left side with those of the right side.

### Ethics statement

This experiment was conducted in accordance with the Declaration of Helsinki (World Medical Association). Informed consent was obtained from the families who participated in the donation program of Southern Medical University and Ningbo University. This study was approved by the Ethics Committee of the Affiliated Hospital of the School of Medicine of Ningbo University.
